# Noninfluenza Vaccination Coverage Among Adults — United States, 2012

**Published:** 2014-02-07

**Authors:** Walter W. Williams, Peng-Jun Lu, Alissa O’Halloran, Carolyn B. Bridges, Tamara Pilishvili, Craig M. Hales, Lauri E. Markowitz

**Affiliations:** 1 Immunization Services Division; 2 Division of Bacterial Diseases; 3 Division of Viral Diseases, National Center for Immunization and Respiratory Diseases; 4 Division of Sexually Transmitted Disease Prevention, National Center for HIV/AIDS, Viral Hepatitis, STD, and TB Prevention, CDC

Vaccinations are recommended throughout life to prevent vaccine-preventable diseases and their sequelae. Adult vaccination coverage, however, remains low for most routinely recommended vaccines (1) and well below *Healthy People 2020* targets.* In October 2013, the Advisory Committee on Immunization Practices (ACIP) approved the adult immunization schedule for 2014 (2). With the exception of influenza vaccination, which is recommended for all adults each year, vaccinations recommended for adults target different populations based on age, health conditions, behavioral risk factors (e.g., injection drug use), occupation, travel, and other indications (2). To assess vaccination coverage among adults aged ≥19 years for selected vaccines, CDC analyzed data from the 2012 National Health Interview Survey (NHIS). This report summarizes the results of that analysis for pneumococcal, tetanus toxoid–containing (tetanus and diphtheria vaccine [Td] or tetanus and diphtheria with acellular pertussis vaccine [Tdap]), hepatitis A, hepatitis B, herpes zoster (shingles), and human papillomavirus (HPV) vaccines by selected characteristics (age, race/ethnicity,† and vaccination target criteria). Influenza vaccination coverage estimates for the 2012–13 influenza season have been published separately (3). Compared with 2011 (1), only modest increases occurred in Tdap vaccination among adults aged 19–64 years, herpes zoster vaccination among adults aged ≥60 years, and HPV vaccination among women aged 19–26 years; coverage among adults in the United States for the other vaccines did not improve. Racial/ethnic gaps in coverage persisted for all six vaccines and widened for Tdap, herpes zoster, and HPV vaccination. Increases in vaccination coverage are needed to reduce the occurrence of vaccine-preventable diseases among adults. The Community Preventive Services Task Force and other authorities have recommended that health-care providers incorporate vaccination needs assessment, recommendation, and offer of vaccination into routine clinical practice for adult patients (4,5).

The NHIS collects information about the health and health care of the noninstitutionalized, civilian population in the United States using nationally representative samples. Interviews are conducted in respondents’ homes by the U.S. Census Bureau for CDC’s National Center for Health Statistics. Questions about receipt of recommended vaccinations for adults are asked of one randomly selected adult within each family in the household. The presence of high-risk conditions,§ as defined by ACIP for pneumococcal disease, was determined by responses to questions in the NHIS (2). Comprehensive information on all high-risk conditions for hepatitis B or A were not collected in the 2012 NHIS. Analyses were conducted to estimate Tdap vaccination of adults aged ≥65 years being collected in the NHIS for the first time starting in 2012. The final sample adult component response rate for the 2012 NHIS was 61.2%. Weighted data¶ were used to produce national vaccination coverage estimates. Point estimates and estimates of corresponding variances were calculated using statistical software to account for the complex sample design. Statistical significance was defined as p<0.05.

## Pneumococcal Vaccination Coverage

Pneumococcal vaccination coverage (overall, for 23-valent pneumococcal polysaccharide vaccine [PPSV23], and for 13-valent pneumococcal conjugate vaccine [PCV13]) among adults aged 19–64 years at high risk was 20.0% overall, similar to the estimate from 2011 (Table 1). Coverage among whites aged 19–64 years at high risk was higher (21.4%) compared with Hispanics (13.8%) and Asians (13.2%), but coverage was not significantly different for blacks and non-Hispanics who indicated a race other than white, black, or Asian. Among adults aged ≥65 years, coverage was 59.9% overall, similar to the estimate for 2011. Coverage among whites aged ≥65 years (64.0%) was higher compared with all other racial/ethnic groups (Table 1).

## Tetanus Vaccination Coverage

In 2012, the proportion of adults receiving any tetanus toxoid–containing vaccine during the past 10 years was 64.2% for adults aged 19–49 years, 63.5% for adults aged 50–64 years, and 55.1% for adults aged ≥65 years (Table 1). The proportion of adults receiving tetanus vaccination during the past 10 years across all age groups did not change compared with 2011 (1). Whites had higher coverage across all age groups compared with blacks, Hispanics, and Asians.

Data on Tdap vaccination of adults aged ≥65 years were collected for the first time in 2012. Among adults aged ≥19 years for whom Tdap vaccination specifically could be assessed (including adults aged ≥65 years), overall coverage was 14.2% (Table 1). Tdap coverage was estimated after excluding from the 34,218 respondents all those for whom Tdap vaccination could not be confirmed, including those without a “yes” or “no” response for tetanus vaccination status in the past 10 years (n = 1,861 [5.4%]) or during 2005–2012 (n = 1,261 [3.7%]), and those who reported tetanus vaccination during 2005–2012 but were not told the vaccine type (n = 6,986 [20.4%]) or did not know the vaccine type (Td or Tdap) (n = 1,457 [4.3%]). Tdap coverage for black (9.8%) and Hispanic (8.7%) adults aged ≥19 years was lower compared with whites (16.1%), but coverage for those who indicated a race other than Asian, black, or white, and non-Hispanic ethnicity was higher (21.4%) than that for whites. Among adults aged 19–64 years, Tdap coverage increased compared with 2011 (a 3.2 percentage point increase to 15.6%) (Table 1); however, coverage among adults aged 19–64 years who reported living with an infant aged <1 year** was 25.9%, similar to the estimate for 2011. Tdap coverage among adults aged 19–64 years without household contact with an infant aged <1 year increased compared with 2011 (a 3.1 percentage point increase to 15.1%). Tdap coverage was higher for whites aged 19–64 years (18.2%) compared with blacks (10.5%) or Hispanics (9.2%). Tdap vaccination coverage among adults aged ≥65 years, overall and among those without household contact with an infant aged <1 year, was 8.0%. The sample was too small to estimate Tdap coverage among adults aged ≥65 years living with an infant aged <1 year. Coverage among Hispanics (3.3%) and Asians (4.2%) aged ≥65 years was lower than for whites (8.8%).

Among 13,145 respondents who received a tetanus vaccination during 2005–2012, 52.6% reported that they were not informed of the vaccination type, and 11.1% could not recall what type of tetanus vaccination they had received (Table 2). Of the remaining 36.3% of respondents who reported they knew what type of tetanus vaccine they received, 65.4% reported receiving Tdap.

During 2005–2012, Tdap vaccination of health-care personnel (HCP) aged ≥19 years was 31.4% (Table 3). White HCP had higher Tdap coverage (33.0%) compared with black HCP (22.5%) and Hispanic HCP (25.1%). Compared with 2011, Tdap coverage increased for HCP aged 19–64 years (by 5.8 percentage points to 32.6%). Tdap coverage among HCP aged ≥65 years was 16.9% (Table 3).

Among adults aged 19–64 years who received a tetanus vaccination and reported they knew what type of tetanus vaccine they received, HCP were more likely to report receipt of Tdap (76.8%) than were non-HCP (64.3%) (Table 2). Tdap vaccination was similar among adults aged ≥65 years who were or were not HCP (Table 2).

## Hepatitis A Vaccination Coverage

Hepatitis A vaccination coverage (≥2 doses) among adults aged 19–49 years was low overall (12.2%), and similar to the estimate for 2011 (12.5%). Coverage was higher for Asians (18.7%) than for whites (12.2%), but coverage for Hispanics (10.5%) was lower than for whites. Vaccination coverage was higher (18.9%) among adults aged 19–49 years who had traveled outside the United States since 1995 to a country of high or intermediate hepatitis A endemicity (countries other than Japan, Australia, New Zealand, Canada, and the countries of Europe) than among respondents who did not travel outside the United States or had traveled only to countries of low endemicity (8.6%). Vaccination coverage among adult travelers to highly endemic countries was similar to the estimate for 2011 (Table 1). Coverage among those with chronic liver conditions could not be reliably estimated because of small sample size.

## Hepatitis B Vaccination Coverage

In 2012, comprehensive information on high-risk status for hepatitis B virus infection was not collected. Overall hepatitis B vaccination coverage (≥3 doses) among all adults aged 19–49 years was 35.3%, similar to the estimate for 2011 (Table 1). Vaccination coverage was lower for blacks (34.2%) and Hispanics (27.1%) compared with whites (37.5%). Vaccination coverage for persons with diabetes was 28.6% for those aged 19–59 years and 15.1% for those aged ≥60 years, similar to the estimates for 2011. Overall, hepatitis B vaccination coverage among HCP was 65.0%, similar to the estimate for 2011, and coverage among HCP did not differ significantly across racial/ethnic groups (Table 3).

## Herpes Zoster Vaccination Coverage

In 2012, 20.1% of adults aged ≥60 years reported receiving herpes zoster vaccination to prevent shingles, an increase from the 15.8% reported in 2011 (Table 1). Coverage for whites aged ≥60 years increased by 5.2 percentage points compared with herpes zoster vaccination coverage estimates in 2011. Whites aged ≥60 years had higher herpes zoster vaccination coverage (22.8%) compared with blacks (8.8%), Hispanics (8.7%), and Asians (16.9%).

## HPV Vaccination Coverage

In 2012, 34.5% of women aged 19–26 years reported receipt of ≥1 dose of HPV vaccine, an increase of 5 percentage points from the 29.5% reported for 2011 (Table 1) (1). Coverage was 44.3% among women aged 19–21 years and 28.2% among those aged 22–26 years (a 6.7 percentage point increase in this age group compared with 2011). Among women aged 19–26 years, blacks (29.1%), Hispanics (18.7%), and Asians (15.6%) had lower coverage compared with whites (42.2%), but coverage for non-Hispanics who indicated a race other than white, black, or Asian was similar to that of whites (41.2%). Receipt of ≥1 dose of HPV vaccine among males aged 19–26 years (2.3%) was similar to the estimate for 2011. Coverage was 2.4% for males aged 19–21 years and 2.2% for those aged 22–26 years.

### Editorial Note

In 2011, adult vaccination coverage in the United States for diseases other than influenza was similar to 2011, except for modest increases in Tdap vaccination for adults aged 19–64 years, herpes zoster vaccination among older adults, and HPV vaccination among women aged 19–26 years, with no improvements in coverage for the other vaccines recommended for adults. Many adults have not received one or more recommended vaccines. Vaccination coverage estimates for the three vaccines in this report that are included in *Healthy People 2020* (pneumococcal, herpes zoster, and hepatitis B [for HCP] vaccines) are well below the respective target levels of 90% for persons aged ≥65 years and 60% for persons aged 18–64 years at high risk (pneumococcal vaccine [objectives IID 13.1 and IID 13.2, respectively]), 30% (herpes zoster vaccine [IID 14]), and 90% (hepatitis B vaccine for HCP [IID 15.3]). In addition, racial/ethnic gaps in coverage persisted for all six and widened for Tdap, herpes zoster, and HPV vaccination, with higher coverage for whites compared with other groups. These data indicate little progress was made in improving adult coverage in the past year and highlight the need for continuing efforts to increase adult vaccination coverage.

In 2006, ACIP recommended that adults aged 19–64 years receive a single dose of Tdap to replace a dose of Td for active booster vaccination against tetanus, diphtheria, and pertussis if they received their most recent dose of Td ≥10 years earlier (6). In 2010, ACIP recommended Tdap, when indicated, should be administered regardless of interval since the last Td, and that adults aged ≥65 years who have or who anticipate having close contact with an infant aged <1 year, and who previously have not received Tdap, should receive a dose of Tdap to protect against pertussis and reduce the likelihood of transmission. In 2011, in an effort to prevent pertussis in infants, ACIP recommended a dose of Tdap for pregnant women who have not yet received a dose, then in 2012, expanded the recommendation for a Tdap dose during every pregnancy. In 2012, ACIP also updated the adult Tdap vaccination recommendation to include all adults aged ≥19 years who have not yet received a dose of Tdap, including those aged ≥65 years (6). Information on Tdap vaccination of adults aged ≥65 years was collected in the 2012 NHIS for the first time. Overall Tdap vaccination of adults aged ≥65 years was low; the sample size was too small to estimate coverage among adults aged ≥65 years living with an infant aged <1 year. Tdap vaccination coverage in 2012 among adults aged ≥65 years could reflect vaccination of those who received Tdap previously because of close contact with an infant aged <1 year, as well as early uptake in response to this recommendation. Health-care providers should not miss an opportunity to vaccinate adults aged ≥19 years who have not received Tdap previously.

In June 2012, ACIP recommended routine use of PCV13 in series with PPSV23 for adults aged ≥19 years with immunocompromising conditions, functional or anatomic asplenia, cerebrospinal fluid leaks, or cochlear implants.†† Given the high burden of invasive pneumococcal disease caused by serotypes in PPSV23, but not in PCV13, ACIP noted that broader protection might be provided through use of both pneumococcal vaccines. Current ACIP recommendations call for use of PPSV23 in adults aged 19–64 years with chronic conditions that are not immunocompromising, such as chronic heart disease or diabetes, at the time of diagnosis of the high-risk condition (6). All adults are eligible for a dose of PPSV23 at age 65 years, regardless of previous PPSV23 or PCV13 vaccination; however, a minimum interval of 5 years between PPSV23 doses should be maintained. The 2012 NHIS did not estimate the proportion of pneumococcal vaccinations by type (PCV13 versus PPSV23). The overall pneumococcal vaccination estimates in this report could include some respondents who received PCV13.

In December 2011, ACIP recommended administration of hepatitis B vaccine to unvaccinated adults with diabetes aged 19–59 years (category A recommendation) or aged ≥60 years (category B recommendation) (6). The recommendations were based on available information about risk for contracting acute hepatitis B among persons with diabetes, morbidity and mortality among persons with diabetes, available vaccines, age at diagnosis of diabetes, and cost-effectiveness (6). Category A recommendations are made for all persons in an age- or risk-factor–based group. Category B recommendations do not apply to all persons within a group, they provide guidance to clinicians to help determine whether vaccination is appropriate for specific patients. Hepatitis B vaccination coverage in 2012 among persons with diabetes remained similar to estimates obtained before this recommendation and highlights the need to improve awareness of increased risk for contracting acute hepatitis B among persons with diabetes and to increase hepatitis B vaccination in this population.

Herpes zoster vaccination coverage increased in 2012 compared with 2011. Shortages of herpes zoster vaccine that might have contributed to lower uptake during the first years after licensure appear to have been resolved in 2012. The cost of herpes zoster vaccine and billing challenges might pose barriers for some patients and providers.§§

The percentage of age-eligible females who reported having received HPV vaccine increased steadily from 2009 to 2012 but is still low. A significant increase in HPV vaccination in 2012 compared with 2011 occurred among women aged 19–26 years (5.0 percentage points). An increase was observed among women aged 22–26 years (6.7 percentage points), but not among women aged 19–21 years. Because no data on age at vaccination were collected, it was not possible to determine whether vaccination occurred as part of an adolescent vaccination program or at age ≥19 years. In 2012, white women had higher HPV coverage than black, Hispanic, or Asian women. Similar findings have been reported previously (7). The percentage of age-eligible adult males administered HPV vaccine in 2012 was similar to the 2011 estimate. Coverage levels for adult males did not change during the first year following the ACIP recommendation for routine use of HPV vaccine in males aged 11–21 years and males aged 22–26 years at high risk (6). However, among adolescent males aged 13–17 years, 2012 HPV coverage estimates were higher than 2011 estimates (8). Continued efforts are needed to ensure coverage among the primary target group for HPV vaccine, girls and boys aged 11–12 years, and among all racial and ethnic groups. Efforts are also needed to improve catch-up vaccination among young adults who have not completed their vaccinations during adolescence.

What is already known on this topic?

During 2008–2011, coverage with routinely recommended vaccinations among U.S. adults aged ≥19 years remained low.

What is added by this report?

Compared with 2011 estimates, modest gains occurred in tetanus and diphtheria toxoid with acellular pertussis vaccine (Tdap) vaccination among adults aged 19–64 years, herpes zoster vaccination among adults aged ≥60 years, and human papillomavirus vaccination coverage among women aged 19–26 years. Coverage for other vaccines and risk groups did not improve, and racial/ethnic disparities persisted for routinely recommended adult vaccines. Coverage for all vaccines for adults remained low.

What are the implications for public health practice?

Wider use of practices shown to improve adult vaccination is needed, including assessment of patients’ vaccination needs by health-care providers and routine recommendation and offer of needed vaccines to adults, implementing reminder-recall systems, use of standing order programs for vaccination, and assessment of practice-level vaccination rates with feedback to staff members.

The findings in this report are subject to at least five limitations. First, the NHIS sample excludes persons in the military and those residing in institutions, which might result in underestimation or overestimation of vaccination coverage levels. Second, the response rate was 61.2%. A low response rate can result in selection bias if the respondents and nonrespondents differ in their vaccination rates. Third, the determination of vaccination status and identification of high-risk conditions in NHIS were not validated by medical records. Self-report of vaccination might be subject to recall bias and overestimation of rates. However, adult self-reported vaccination status has been shown to be sensitive for all six vaccines in this report and specific for all except tetanus vaccination (9). Fourth, the Tdap estimate is subject to considerable uncertainty. Many respondents were excluded from estimations of Tdap coverage, creating a potential for bias. All respondents who reported a tetanus vaccination during 2005–2012 but were unable to say whether Td or Tdap was used were excluded. Sensitivity calculations were conducted to assess the magnitude of potential bias. Depending on what proportion of excluded respondents actually received Tdap, actual Tdap coverage could fall within the range of 11.2%–39.4% for adults aged 19–64 years and 6.0%–31.0% for adults aged ≥65 years. Comparisons of Tdap coverage across years within subgroups might be affected by bias resulting from excluding persons who did not report the type of tetanus vaccine they received. Finally, age at vaccination is not known for vaccines adults reported having “ever” received (e.g., HPV and hepatitis B vaccines), so it is not clear for younger adults whether vaccination occurred as an adult or as part of a child or adolescent vaccination program.

Vaccination coverage levels among adults are low. Improvement in adult vaccination is needed to reduce the health consequences of vaccine-preventable diseases among adults and to prevent pertussis morbidity and mortality in infants, who need the protection afforded by the Tdap vaccination during pregnancy recommendation. Successful vaccination programs combine 1) education of potential vaccine recipients and publicity to promote vaccination, 2) increased access to vaccination services in medical settings, and 3) use of practices shown to improve vaccination coverage, including reminder-recall systems, efforts to remove administrative and financial barriers to vaccination, use of standing order programs for vaccination, and assessment of practice-level vaccination rates with feedback to staff members (4). Health-care provider recommendations for vaccination are associated with patient vaccination (10). Routine assessment of adult patient vaccination needs, recommendation, and offer of needed vaccinations for adults should be incorporated into routine clinical care of adults (4,5). The adult immunization schedule (2), updated annually, provides current recommendations for vaccinating adults and a ready resource for persons who provide health-care services for adults in various settings.

## Figures and Tables

**TABLE 1:** Estimated proportion of adults aged ≥19 years who received selected vaccinations, by age group, high-risk status,* race/ethnicity, and other selected characteristics — National Health Interview Survey, United States, 2012

Vaccination, age group, high-risk status, and race/ethnicity†	Sample size	%	(95% CI)	Difference from 2011
**Pneumococcal vaccination, ever** §				
19–64 yrs, high risk				
Total	9,333	20.0	(18.9–21.1)	−0.1
White	5,736	21.4	(20.1–22.9)	1.3
Black	1,605	19.7	(17.4–22.2)	−3.1
Hispanic	1,326	13.8	(11.5–16.4)¶	−4.6**
Asian	350	13.2	(9.5–18.1)¶	1.2
Others	316	20.2	(15.2–26.2)	−1.5
≥65 yrs				
Total	7,076	59.9	(58.4–61.4)	−2.4
White	4,993	64.0	(62.3–65.7)	−2.5
Black	919	46.1	(41.7–50.6)¶	−1.5
Hispanic or Latino	698	43.4	(39.0–48.0)¶	0.3
Asian	373	41.3	(35.4–47.5)¶	1.0
Others	93	44.7	(32.6–57.5)¶	−22.7**
**Tetanus vaccination, past 10 yrs** ††				
19–49 yrs				
Total	16,927	64.2	(63.2–65.1)	−0.3
White	8,969	69.7	(68.5–70.9)	0.1
Black	2,491	56.1	(53.5–58.6)¶	1.3
Hispanic	3,772	53.9	(51.9–56.0)¶	−2.4
Asian	1,195	54.3	(50.6–58.0)¶	1.9
Others	500	71.9	(66.5–76.8)	2.3
50–64 yrs				
Total	8,525	63.5	(62.1–64.8)	−0.4
White	5,577	67.5	(65.9–69.0)	−0.2
Black	1,373	52.3	(49.0–55.7)¶	−2.1
Hispanic	1,031	52.3	(47.8–56.8)¶	−0.3
Asian	371	48.2	(41.8–54.7)¶	3.1
Others	173	69.9	(60.3–78.0)	2.0
≥65 yrs				
Total	6,905	55.1	(53.6–56.7)	0.7
White	4,864	57.7	(55.9–59.5)	0.8
Black	904	44.6	(40.8–48.4)¶	0.2
Hispanic	678	44.8	(40.1–49.6)¶	−0.3
Asian	366	45.8	(39.5–52.2)¶	7.9
Others	93	50.2	(36.8–63.6)	−13.0
**Tetanus vaccination including pertussis vaccine, past 7 yrs** §§				
≥19 yrs				
Total	22,653	14.2	(13.6–14.9)	NA
White	13,135	16.1	(15.3–17.0)	NA
Black	3,434	9.8	(8.4–11.6)¶	NA
Hispanic	4,051	8.7	(7.6–10.0)¶	NA
Asian	1,526	14.7	(12.5–17.2)	NA
Others	507	21.4	(17.0–26.7)¶	NA
Living with an infant aged <1 yr	722	25.9	(22.4–29.8)	NA
Not living with an infant aged <1 yr	21,931	13.8	(13.2–14.5)	NA
19–64 yrs				
Total	17,695	15.6	(14.9–16.4)	3.2**
White	9,729	18.2	(17.2–19.2)	4.4**
Black	2,746	10.5	(8.9–12.3)¶	−0.5
Hispanic	3,544	9.2	(8.0–10.6)¶	1.5
Asian	1,237	16.2	(13.8–19.0)	4.5**
Others	439	22.7	(17.8–28.5)	3.0
Living with an infant aged <1 yr	716	25.9	(22.3–29.8)	4.4
Not living with an infant aged <1 yr	16,979	15.1	(14.4–15.9)	3.1**
≥65 yrs				
Total	4,958	8.0	(7.0–9.1)	NA
White	3,406	8.8	(7.6–10.2)	NA
Black	688	5.9	(3.7–9.4)	NA
Hispanic	507	3.3	(2.0–5.4)¶	NA
Asian	289	4.2	(2.4–7.3)¶	NA
Others	68	—¶¶		NA
Living with an infant aged <1 yr	6	—¶¶		NA
Not living with an infant aged <1 yr	4,952	8.0	(7.0–9.1)	NA
**Hepatitis A vaccination (≥2 doses), ever** ***				
19–49 yrs				
Total	14,834	12.2	(11.5–13.0)	−0.3
White	7,887	12.2	(11.2–13.2)	−0.1
Black	2,207	11.3	(9.6–13.2)	0.1
Hispanic	3,341	10.5	(9.2–11.9)¶	−0.8
Asian	992	18.7	(15.7–22.1)¶	−0.4
Others	407	16.1	(11.4–22.2)	−5.0
Had traveled outside the United States since 1995, other than to Europe, Japan, Australia, New Zealand, or Canada	5,259	18.9	(17.6–20.3)	−1.2
Had not traveled outside the United States since 1995, other than to Europe, Japan, Australia, New Zealand, or Canada	9,548	8.6	(7.8–9.5)	0.2
With chronic liver conditions, overall	121	—¶¶		—¶¶
**Hepatitis B vaccination (≥3 doses), ever** †††				
19–49 yrs				
total	15,649	35.3	(34.3–36.2)	−0.7
white	8,296	37.5	(36.3–38.8)	−0.3
black	2,338	34.2	(31.5–36.9)¶	1.2
Hispanic	3,465	27.1	(25.1–29.2)¶	−1.8
Asian	1,105	39.7	(35.5–44.0)	−0.9
others	445	37.4	(31.9–43.3)	−6.7
With diabetes				
Overall	1,286	28.6	(25.4–32.1)	1.7
≥60 yrs, overall	1,907	15.1	(12.9–17.4)	2.6
**Herpes Zoster (shingles) vaccination, ever** §§§				
≥60 yrs				
Total	9,924	20.1	(19.1–21.2)	4.4**
White	6,957	22.8	(21.5–24.0)	5.2**
Black	1,354	8.8	(6.9–11.2)¶	0.9
Hispanic	990	8.7	(6.6–11.4)¶	0.7
Asian	487	16.9	(13.2–21.5)¶	3.0
Others	136	19.7	(11.5–31.6)	7.7
**Human papillomavirus (HPV) vaccination among females (≥1 dose), ever** ¶¶¶				
19–26 yrs				
Total	2,300	34.5	(31.7–37.3)	5.0**
White	1,165	42.2	(38.5–46.0)	9.7**
Black	385	29.1	(23.4–35.7)¶	0.9
Hispanic	507	18.7	(14.9–23.1)¶	−1.5
Asian	148	15.6	(9.5–24.5)¶	−6.7
Others	95	41.2	(28.7–55.0)	2.2
19–21 yrs, total	760	44.3	(39.5–49.2)	1.2
22–26 yrs, total	1,540	28.2	(25.2–31.5)	6.7**
**Human papillomavirus (HPV) vaccination among males (≥1 dose), ever** ¶¶¶				
19–26 yrs, total	1,783	2.3	(1.6–3.4)	0.2
19–21 yrs, total	634	2.4	(1.4–4.4)	−0.3
22–26 yrs, total	1,149	2.2	(1.3–3.8)	0.5

* Adults were considered at high risk for pneumococcal disease or its complications if they had ever been told by a doctor or other health professional that they had diabetes, emphysema, chronic obstructive pulmonary disease, coronary heart disease, angina, heart attack, or other heart condition; had a diagnosis of cancer during the previous 12 months (excluding nonmelanoma skin cancer); had ever been told by a doctor or other health professional that they had lymphoma, leukemia, or blood cancer; had been told by a doctor or other health professional that they had chronic bronchitis or weak or failing kidneys during the preceding 12 months; had an asthma episode or attack during the preceding 12 months; or were current smokers. Comprehensive information on high-risk conditions for hepatitis B or A was not collected in 2012.

† Race/ethnicity was categorized as Hispanic, black, white, Asian, and “other.” Persons identified as Hispanic might be of any race. Persons identified as black, white, Asian, or other race are non-Hispanic. “Other” includes American Indian/Alaska Native and multiple race. The five racial/ethnic categories are mutually exclusive.

§ Respondents were asked if they had ever had a pneumonia shot.

¶ p<0.05 by t-test for comparisons, with non-Hispanic white as the reference.

** p<0.05 by t-test for comparisons between 2012 and 2011 within each level of each characteristic.

†† Respondents were asked if they had received a tetanus shot in the past 10 years. Vaccinated respondents included adults who received tetanus-diphthera toxoid vaccine (Td) during the past 10 years or tetanus, diphthera, and acellular pertussis vaccine (Tdap) during 2005–2012.

§§ Respondents who had received a tetanus shot in the past 10 years were asked if their most recent shot was given in 2005 or later. Respondents who had received a tetanus shot since 2005 were asked if they were told that their most recent tetanus shot included the pertussis or whooping cough vaccine. Among 34,218 respondents aged ≥19 years, those without a “yes” or no” classification for tetanus vaccination status within the preceding 10 years (n = 1,861 [5.4%]), for tetanus vaccination status during 2005–2012 (n = 1,261 [3.7%]), or those who reported tetanus vaccination during 2005–2012, but were not told vaccine type by the provider (n = 6,986 [20.4%] or did not know vaccine type (Td or Tdap) (n = 1,457 [4.3%]) were excluded, yielding a sample of 22,653 respondents aged ≥19 years for whom Tdap vaccination status could be assessed. In February 2012, the Advisory Committee on Immunization Practices recommended Tdap vaccination for all adults aged ≥19 years, including adults aged ≥65 years.

¶¶ Estimate is not reliable because of small sample size (<30) or relative standard error (standard error/estimates) >0.3.

*** Respondents were asked if they had ever received the hepatitis A vaccine, and if yes, were asked how many shots were received.

††† Respondents were asked if they had ever received the hepatitis B vaccine, and if yes, if they had received ≥3 doses or <3 doses.

§§§ Respondents were asked if they had ever received a shingles vaccine.

¶¶¶ Respondents were asked if they had ever received the HPV shot or cervical cancer vaccine.

**TABLE 2:** Type of tetanus vaccine received, and proportion that were tetanus, diphtheria, acellular pertussis vaccine (Tdap), among adults aged ≥19 years who received a tetanus vaccination, by selected characteristics — National Health Interview Survey, United States, 2005–2012

	Type of vaccine received											
		
		Received Tdap		Received other tetanus vaccine		Doctor did not inform the patient		Could not recall vaccine type		Proportion that received Tdap*		
						
Characteristic	No. in sample	%	(95% CI)	%	(95% CI)	%	(95% CI)	%	(95% CI)	No. in sample	%	(95% CI)
**≥19 yrs**												
All adults	13,145	23.8	(22.7–24.9)	12.6	(11.8–13.4)	52.6	(51.2–54.0)	11.1	(10.2–12.0)	4,699	65.4	(63.5–67.3)
Health-care personnel†	1,501	44.0	(40.2–47.8)	13.7	(11.4–16.3)	33.1	(29.8–36.6)	9.3	(7.5–11.4)	857	76.3§	(72.0–80.1)
Non–health-care personnel	11,631	21.2	(20.2–22.3)	12.4	(11.6–13.3)	55.1	(53.6–56.5)	11.3	(10.4–12.2)	3,840	63.1	(61.0–65.1)
**19–64 yrs**												
All adults	10,932	24.9	(23.8–26.1)	12.5	(11.6–13.3)	51.5	(50.0–53.1)	11.1	(10.1–12.0)	4,065	66.7	(64.7–68.6)
Health-care personnel	1,394	44.8	(40.9–48.8)	13.5	(11.2–16.2)	32.7	(29.3–36.3)	8.9	(7.1–11.2)	809	76.8§	(72.5–80.6)
Non–health-care personnel	9,527	22.2	(21.0–23.3)	12.3	(11.4–13.3)	54.2	(52.6–55.8)	11.3	(10.3–12.4)	3,254	64.3	(62.0–66.4)
**≥65 yrs**												
All adults	2,213	16.8	(14.8–19.0)	13.1	(11.4–15.1)	59.0	(56.2–61.7)	11.1	(9.6–12.8)	634	56.1	(50.8–61.2)
Health-care personnel	107	30.1	(19.7–43.0)	16.2	(9.1–27.2)	39.0	(27.9–51.3)	14.7	(7.9–25.8)	48	65.0	(46.5–79.9)
Non–health-care personnel	2,104	16.2	(14.1–18.5)	13.0	(11.2–15.1)	59.9	(56.9–62.8)	10.9	(9.3–12.6)	586	55.4	(50.0–60.7)

* Calculated by dividing number of respondents who reported receiving Tdap by the sum of those who reported receiving Tdap and those who reported receiving other tetanus vaccinations. Respondents who reported that the doctor did not inform them of the vaccine type they received and those who could not recall the vaccine type were excluded.

† Adults were classified as health-care personnel if they reported they currently volunteer or work in a hospital, medical clinic, doctor’s office, dentist’s office, nursing home, or other health-care facility, including part-time and unpaid work in a health-care facility or professional nursing care provided in the home.

§ p<0.05 by t-test for comparisons between health-care personnel and non–health-care personnel.

**TABLE 3:** Estimated proportion of health-care personnel* who received selected vaccinations, by age group and race/ethnicity† — National Health Interview Survey, United States, 2012

Vaccination status	Sample size	%	(95% confidence Interval)	Difference from 2011
**Tetanus vaccination including pertussis vaccine, past 7 years** §				
≥19 yrs				
Total	2,105	31.4	(28.7–34.3)	NA
White	1,262	33.0	(29.5–36.7)	NA
Black	359	22.5	(17.4–28.5)¶	NA
Hispanic	262	25.1	(19.0–32.3)¶	NA
Asian	169	39.4	(30.2–49.5)	NA
Other	53	46.1	(27.7–65.7)	NA
19–64 yrs				
Total	1,911	32.6	(29.7–35.6)	5.8**
White	1,123	34.5	(30.7–38.5)	7.3**
Black	337	22.9	(17.7–29.1)¶	1.3
Hispanic	247	25.1	(18.8–32.7)¶	−4.9
Asian	154	41.4	(31.7–51.8)	13.7
Others	50	46.1	(27.2–66.1)	14.8
≥65 yrs				
Total	194	16.9	(11.3–24.6)	NA
White	139	17.5	(11.0–26.8)	NA
Black	22	—††	—	NA
Hispanic or Latino	15	—††	—	NA
Asian	15	—††	—	NA
Other	3	—††	—	NA
**Hepatitis B vaccination (≥3 doses), ever** §§				
≥19 yrs				
Total	2,767	65.0	(62.7–67.2)	1.1
White	1,692	65.5	(62.5–68.4)	0.4
Black	479	61.7	(56.4–66.7)	4.6
Hispanic	332	60.1	(53.1–66.7)	0.6
Asian	195	72.3	(63.4–79.7)	1.9
Other	69	75.9	(62.2–85.7)	5.9

**Abbreviation:** NA = not available.

* Adults were classified as health-care personnel if they reported that they currently volunteer or work in a hospital, medical clinic, doctor’s office, dentist’s office, nursing home, or other health-care facility, including part-time and unpaid work in a health-care facility or professional nursing care provided in the home.

† Race/ethnicity was categorized as Hispanic, black, white, Asian, and “other.” Persons identified as Hispanic might be of any race. Persons identified as black, white, Asian, or other race are non-Hispanic. “Other” includes American Indian/Alaska Native and multiple race. The five racial/ethnic categories are mutually exclusive.

§ Respondents who had received a tetanus shot in the past 10 years were asked if their most recent shot was given in 2005 or later. Respondents who had received a tetanus shot since 2005 were asked if they were told that their most recent tetanus shot included the pertussis or whooping cough vaccine. Among 2,911 health-care personnel aged ≥19 years, those without a “yes” or “no” classification for tetanus vaccination status within the preceding 10 years (n = 63 [2.2%]) for tetanus vaccination status during 2005–2012 (n = 100 [3.4%]) or those who reported tetanus vaccination during 2005–2012, but who were not told vaccine type by the provider (n = 516 [17.7%]) or did not know vaccine type (tetanus and diphtheria vaccine [Td] or tetanus and diphtheria with acellular pertussis vaccine [Tdap]) (n = 127 [4.4%]) were excluded, yielding a sample of 2,105 respondents aged ≥19 years for whom Tdap vaccination status could be assessed. In February 2012, the Advisory Committee on Immunization Practices recommended Tdap vaccination for all adults aged ≥19 years, including adults aged ≥65 years.

¶ p<0.05 by t-test for comparisons with non-Hispanic white as the reference.

** p<0.05 by t-test for comparisons between 2012 and 2011 within each level of each characteristic.

†† Estimate is not reliable because of small sample size (<30) or relative standard error (standard error/estimates) >0.3.

§§ Respondents were asked if they had ever received the hepatitis B vaccine, and if yes, if they had received ≥3 doses or <3 doses.

## Footnotes

* *Healthy People 2020* objectives and targets for immunization and infectious diseases are available at http://www.healthypeople.gov/2020/topicsobjectives2020/objectiveslist.aspx?topicid=23.

† Race/ethnicity was categorized as Hispanic, black, white, Asian, and “other.” Persons identified as Hispanic might be of any race. Persons identified as black, white, Asian, or other race are non-Hispanic. “Other” includes American Indian/Alaska Native and multiple race. The five racial/ethnic categories are mutually exclusive.

§ Adults were considered at high risk for pneumococcal disease or its complications if they had ever been told by a doctor or other health professional that they had diabetes, emphysema, chronic obstructive pulmonary disease, coronary heart disease, angina, heart attack, or other heart condition; had a diagnosis of cancer during the previous 12 months (excluding nonmelanoma skin cancer); had ever been told by a doctor or other health professional that they had lymphoma, leukemia, or blood cancer; had been told by a doctor or other health professional that they had chronic bronchitis or weak or failing kidneys during the preceding 12 months; had an asthma episode or attack during the preceding 12 months; or were current smokers.

¶ Additional information on NHIS methods is available at http://www.cdc.gov/nchs/nhis/methods.htm.

** In 2006, a single dose of Tdap was recommended for adults who have or who anticipate having close contact with an infant aged <1 year (e.g., parents, grandparents, child-care providers, and health-care personnel) to reduce the risk for transmitting pertussis.

†† Additional information available at http://www.cdc.gov/mmwr/preview/mmwrhtml/mm6140a4.htm?s_cid=mm6140a4_w.

§§ Additional information available at http://www.gao.gov/assets/590/587009.pdf.

## References

[1] CDC (2013). Noninfluenza vaccination coverage among adults—United States, 2011. MMWR.

[2] CDC (2014). Advisory Committee on Immunization Practices recommended immunization schedule for adults aged 19 years and older—United States, 2014. MMWR.

[3] CDC (2013). Flu vaccination coverage, United States, 2012–13 influenza season.

[4] Community Preventive Services Task Force (2011). The guide to community preventive services Increasing appropriate vaccination: universally recommended vaccinations.

[5] Poland GA, Shefer AM, McCauley M (2003). Standards for adult immunization practices. Am J Prev Med.

[6] CDC (2012). Advisory Committee on Immunization Practices (ACIP) recommendations.

[7] Williams WW, Lu PJ, Saraiya M (2013). Factors associated with human papillomavirus vaccination among young adult women in the United States. Vaccine.

[8] CDC (2013). National and state vaccination coverage among adolescents aged 13–17 years—United States, 2012. MMWR.

[9] Rolnick SJ, Parker ED, Nordin JD (2013). Self-report compared to electronic medical record across eight adult vaccines: do results vary by demographic factors?. Vaccine.

[10] Winston CA, Wortley PM, Lees KA (2006). Factors associated with vaccination of Medicare beneficiaries in five U.S. communities: results from the racial and ethnic adult disparities in immunization initiative survey, 2003. J Am Geriatr Soc.

